# Supplementary material for: Multi-Environment Model Estimation for Motility Analysis of Caenorhabditis elegans
Source: PLoS One. 2010 Jul 22;5(7):e11631. doi: 10.1371/journal.pone.0011631 (PMC2908547; doi:10.1371/journal.pone.0011631)
Supplement: Table S1 — Compiled data on segmentation results for the Multi-Environment Model Estimation (MEME) and threshold-based algorithms. Performances of each algorithm (i.e., surface error and nematode yield) are evaluated for 13 different image sequences representative of various locomotive environments (e.g., crawling on agar plate, swimming in a channel or a drop, locomotion in microfluidic substrates). (0.37 MB PDF) [file pone.0011631.s001.pdf]

Supplementary Table 1

Motility Assay	Image (pix^2)	Worm Area (% image)	Method	Threshold (intensity)	Surface Error (%)	Nematode Yield (%)
Crawl 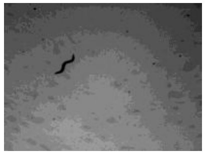 (*n*=36)	480 x 640	0.48 ± 0.02	Threshold	85	0.165 ± 0.002	68.83 ± 0.31
			Threshold	90	0.160 ± 0.002	71.60 ± 0.30
			Threshold	93	0.160 ± 0.002	73.68 ± 0.28
			Threshold	95	0.259 ± 0.003	75.29 ± 0.25
			**Model**	N/A	**0.178 ± 0.003**	**83.81 ± 0.40**
Drop 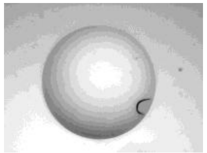 (*n*=36)	480 x 640	0.39 ± 0.02	Threshold*	25	0.176 ± 0.003	57.80 ± 0.53
			Threshold*	20	0.171 ± 0.002	62.17 ± 0.59
			Threshold*	17	0.174 ± 0.002	65.67 ± 0.67
			Threshold*	15	0.180 ± 0.002	68.95 ± 0.72
			Threshold*	10	0.194 ± 0.003	73.28 ± 0.72
			**Model**	N/A	**0.191 ± 0.003**	**79.43 ± 0.51**

* with background subtraction

red – optimized threshold

Supplementary Table 1 (continued)

Motility Assay	Image (pix^2)	Worm Area (% image)	Method	Threshold (intensity)	Surface Error (%)	Nematode Yield (%)
Drops II 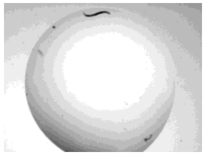 (*n*=36)	480 x 640	0.43 ± 0.03	Threshold*	30	0.218 ± 0.004	50.18 ± 0.52
			Threshold*	25	0.207 ± 0.004	52.76 ± 0.52
			Threshold*	20	0.202 ± 0.008	55.63 ± 0.55
			**Model**	N/A	**0.178 ± 0.004**	**61.13 ± 0.58**
Channel 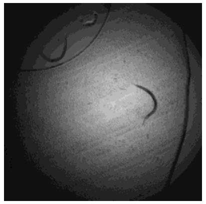 (*n*=36)	1024 x 1024	0.45 ± 0.02	Threshold*	30	0.094 ± 0.002	81.75 ± 0.43
			Threshold*	25	0.084 ± 0.002	85.01 ± 0.43
			Threshold*	20	0.076 ± 0.002	88.04 ± 0.41
			Threshold*	15	0.071± 0.002	90.76 ± 0.40
			Threshold*	10	0.069 ± 0.002	93.64 ± 0.34
			Threshold*	10	0.078 ± 0.002	95.49 ± 0.28
			**Model**	N/A	**0.082 ± 0.002**	**89.85 ± 0.41**

* with background subtraction

red – optimized threshold

Supplementary Table 1 (continued)

Motility Assay	Image (pix^2)	Worm Area (% image)	Method	Threshold (intensity)	Surface Error (%)	Nematode Yield (%)
Channel II	1024 x 1024	0.48 ± 0.02	Threshold*	30	0.151 ± 0.005	74.26 ± 0.67
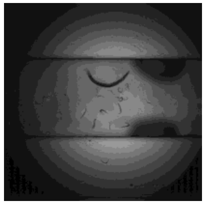			Threshold*	25	0.132 ± 0.005	78.43 ± 0.68
			Threshold*	20	0.115 ± 0.004	82.56 ± 0.28
			Threshold*	15	0.102 ± 0.004	86.66 ± 0.62
(*n*=37)			Threshold*	20	0.155 ± 0.003	78.54 ± 0.51
			**Model**	N/A	**0.100 ± 0.003**	**89.57 ± 0.06**
Micro-fluidic #	176 x 221	5.11 ± 0.28	Threshold	90	3.649 ± 0.045	28.97 ± 0.31
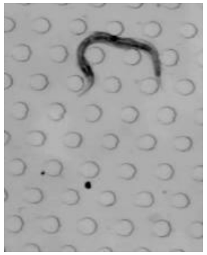			Threshold	100	3.384 ± 0.041	34.38 ± 0.30
			Threshold	110	3.124 ± 0.039	39.94 ± 0.32
			Threshold	120	2.907 ± 0.039	44.74 ± 0.33
			Threshold	125	4.628 ± 0.040	47.19 ± 0.36
(*n*=37)			**Model**	N/A	**2.241 ± 0.028**	**77.83 ± 0.50**

S.R. Lockery *et al.*, Artificial Dirt: Microfluidic Substrates for Nematode Neurobiology and Behavior, *J. Neurophysiol.* 99: 3136 (2008); Suppl. Material (Video 1)

* with background subtraction

red – optimized threshold

Supplementary Table 1 (continued)

Motility Assay	Image (pix²)	Worm Area (% image)	Method	Threshold (intensity)	Surface Error (%)	Nematode Yield (%)
Micro-fluidic II # 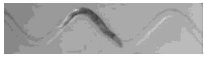 (*n*=37)	301 x 77	7.65 ± 0.58	Threshold	100	7.040 ± 0.149	8.21 ± 1.29
			Threshold	110	5.775 ± 0.096	24.80 ± 0.45
			Threshold	120	4.963 ± 0.084	35.87 ± 0.51
			Threshold	130	5.454 ± 0.024	29.36 ± 3.57
			**Model**	N/A	**3.370 ± 0.082**	**62.55 ± 1.00**
Micro-fluidic III # 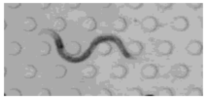 (*n*=36)	295 x 137	7.52 ± 0.31	Threshold	120	4.659 ± 0.062	40.05 ± 0.30
			Threshold	130	4.116 ± 0.047	46.43 ± 0.34
			Threshold	140	3.713 ± 0.042	53.25 ± 0.35
			Threshold	150	3.354 ± 0.035	61.70 ± 0.37
			Threshold	160	3.537 ± 0.033	70.97 ± 0.34
			Threshold	170	7.162 ± 0.078	80.10 ± 2.29
			**Model**	N/A	**3.456 ± 0.036**	**56.98 ± 0.59**

S.R. Lockery *et al.*, Artificial Dirt: Microfluidic Substrates for Nematode Neurobiology and Behavior, *J. Neurophysiol.* 99: 3136 (2008); Suppl. Material (Videos 2 & 3)

red – optimized threshold

Supplementary Table 1 (continued)

Motility Assay	Image (pix²)	Worm Area (% image)	Method	Threshold (intensity)	Surface Error (%)	Nematode Yield (%)
Swim [$]	320 x 240	0.65 ± 0.06	Threshold*	25	0.127 ± 0.006	87.09 ± 0.94
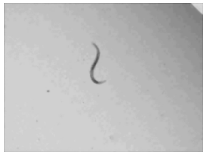			Threshold*	20	0.123 ± 0.005	91.46 ± 0.81
			Threshold*	15	0.134 ± 0.007	95.37 ± 0.61
(*n*=31)			Threshold*	10	0.182 ± 0.007	98.15± 0.38
			Threshold*	7	0.291 ± 0.022	92.93 ± 4.39
			**Model**	N/A	**0.256 ± 0.007**	**91.94 ± 0.58**
Swim II [$]	320 x 240	0.82 ± 0.03	Threshold	130	0.129 ± 0.003	86.27 ± 0.36
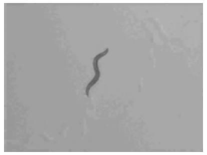			Threshold	140	0.100 ± 0.003	94.71 ± 0.25
			Threshold	150	0.153 ± 0.004	98.79 ± 0.14
(*n*=38)			Threshold	160	0.463 ± 0.017	94.65 ± 3.62
			**Model**	N/A	**0.278 ± 0.004**	**98.81 ± 0.09**

[$] J. Pierce-Shimonura *et al.*, Genetic analysis of crawling and swimming locomotory patterns in *C. elegans*, *Proc. Natl. Acad. Sci. USA* 105: 20982–20987 (2008); Suppl. Material (SM2.avi; SM5.avi)

* with background subtraction

red – optimized threshold

Supplementary Table 1 (continued)

**Motility Assay**	**Image (pix^2)**	**Worm Area (% image)**	**Method**	**Threshold (intensity)**	**Surface Error (%)**	**Nematode Yield (%)**
Gelatin [+] 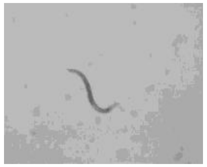 (*n*=29)	700 x 567	1.30 ± 0.05	Threshold	100	1.203 ± 0.007	57.32 ± 0.29
			Threshold	110	1.102 ± 0.006	66.59 ± 0.33
			Threshold	110	0.681 ± 0.005	74.50 ± 0.33
			Threshold	120	0.375 ± 0.005	82.01 ± 0.30
			Threshold	130	0.513 ± 0.048	87.11 ± 3.12
			**Model**	N/A	**0.482 ± 0.082**	**85.91 ± 0.22**
Gelatin II 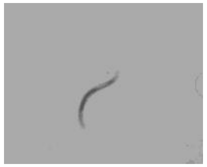 (*n*=25)	700 x 567	1.49 ± 0.08	Threshold	130	1.405 ± 0.015	48.98 ± 0.51
			Threshold	140	1.264 ± 0.014	58.97 ± 0.54
			Threshold	150	0.490 ± 0.008	69.65 ± 0.57
			Threshold	155	0.431 ± 0.008	75.50 ± 0.52
			Threshold	160	0.447 ± 0.054	81.97 ± 0.44
			**Model**	N/A	**0.472 ± 0.007**	**85.78 ± 0.58**

[+] S. Berry *et al.*, Forward locomotion of the nematode *C. elegans* is achieved through modulation of a single gait, *HFSP J.* 3:186–193 (2009); Suppl. Material (berri_gel_1.2_perc.mov; berri_gel_0.0_perc.mov)

red – optimized threshold

Supplementary Table 1 (continued)

**Motility Assay**	**Image (pix²)**	**Worm Area (% image)**	**Method**	**Threshold (intensity)**	**Surface Error (%)**	**Nematode Yield (%)**
Gelatin III [+] 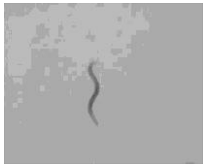 (*n*=33)	700 x 567	1.51 ± 0.08	Threshold	140	1.354 ± 0.012	53.33 ± 0.42
			Threshold	150	1.237 ± 0.011	61.98 ± 0.46
			Threshold	155	0.543 ± 0.010	66.53 ± 0.50
			Threshold	160	0.489 ± 0.009	71.35 ± 0.49
			Threshold	170	0.486 ± 0.010	84.31 ± 0.36
			**Model**	N/A	**0.496 ± 0.007**	**82.94 ± 0.45**

[+] S. Berry *et al.*, Forward locomotion of the nematode *C. elegans* is achieved through modulation of a single gait, *HFSP J.* 3:186–193 (2009); Suppl. Material (berri_gel_2.0_perc.mov)

red – optimized threshold
